# Promising predictors of diabetic peripheral neuropathy in children and adolescents with type 1 diabetes mellitus

**DOI:** 10.1186/s13052-024-01774-y

**Published:** 2024-10-14

**Authors:** Ahmed S. Abo Hola, Sameh A. Abd El Naby, Esraa T. Allam, Ayaat A. Gab Allah, Dina A. Hammad

**Affiliations:** 1 Department of Pediatrics, Faculty of Medicine, Menoufia University, Yassin Abdel-Ghafar Street, Shebin El-Kom, Shebin El-Kom, 32511 Menoufia Egypt; 2 Department of Clinical Pathology, National Liver Institute, Menoufia University, Shebin El-Kom, Egypt

**Keywords:** Diabetic peripheral neuropathy, Heat shock protein 27, Lipid profile, Michigan neuropathy screening instrument, Neuron specific enolase, Type 1 diabetes mellitus

## Abstract

**Background:**

Diabetic peripheral neuropathy (DPN) in children and adolescents with type 1 diabetes mellitus (T1DM) is a growing issue, with controversial data in the terms of prevalence and evaluation timelines. Currently, there are no clear standards for its early detection. Therefore, our aim was to assess the contribution of the Michigan neuropathy screening instrument (MNSI), lipid profile, serum neuron specific enolase (NSE), and serum heat shock protein 27 (HSP 27) to the prediction of DPN in children and adolescents with T1DM.

**Methods:**

In this case-control study, fifty children diagnosed with T1DM for at least five years were enrolled and evaluated through complete neurological examination, MNSI, and nerve conduction study (NCS). Additionally, HbA1c, lipid profile, serum NSE, and serum HSP 27 levels were measured for patients and controls.

**Results:**

The prevalence of DPN in our study was 24% by NCS, and electrophysiological changes showed a statistically significant lower conduction velocity for the posterior tibial and sural nerves, as well as a prolonged latency period for the common peroneal and sural nerves in neuropathic patients. In these patients, older age, earlier age of diabetes onset, longer disease duration, higher total cholesterol, triglycerides, low density lipoprotein cholesterol, HbA1c, serum NSE, and HSP27 levels were observed. The MNSI examination score ≥ 1.5 cutoff point had an area under the curve (AUC) of 0.955, with 75% sensitivity and 94.74% specificity, according to receiver operating characteristic curve analysis. However, the questionnaire’s cutoff point of ≥ 5 had an AUC of 0.720, 75% sensitivity, and 63% specificity, with improved overall instrument performance when combining both scores. Regarding blood biomarkers, serum NSE had greater sensitivity and specificity in discriminating neuropathic patients than HSP27 (92% and 74% versus 75% and 71%, respectively). Regression analysis revealed a substantial dependency for MNSI and serum NSE in predicting DPN in patients.

**Conclusions:**

Despite limited research in pediatrics, MNSI and serum NSE are promising predictive tools for DPN in children and adolescents with T1DM, even when they are asymptomatic. Poor glycemic control and lipid profile changes may play a critical role in the development of DPN in these patients, despite conflicting results in various studies.

## Background

Among children and adolescents, type 1 diabetes mellitus (T1DM) is one of the most common chronic diseases, representing nearly 98% of diabetes cases underage of 10 years and 87% in adolescents with diabetes. Therefore, diabetic peripheral neuropathy (DPN) in children and adolescents is becoming more prevalent especially with early age of onset, longer lifetime, and poor glycemic control [1–3]. The American Diabetes Association (ADA) recommended that all patients with T1DM should be assessed for distal peripheral neuropathy (PN) 5 years following diagnosis, and at least annually after that. Nevertheless, the reported prevalence of DPN in children and adolescents with T1DM is scarce among different studies and ranges from 3 to 62%, which may be owed to racial and ethnic differences and the variability of methods used in characterizing and diagnosing neuropathy [4–6].

Clinically apparent presentation of DPN is uncommon in pediatric populations; still, the development of DPN may begin early during diabetes course, and subclinical autonomic neuropathy may develop within the first two years of T1DM diagnosis and within a year in type 2 diabetes mellitus (T2DM) [3, 7]. The nerve conduction study (NCS) is the gold standard test for diagnosing PN by measuring an individual’s peripheral nerve’s capacity to send electrical signals, however, the test is costly, time-consuming, and necessitates professional expertise [8]. To overcome these obstacles, a few clinical grading systems have been created, including Michigan neuropathy screening instrument (MNSI), which is a low-cost, non-invasive, and simple-to-use tool that both patients and doctors can employ to evaluate possibility of PN [9]. Despite this, there is still insufficient data about its significance in children.

Dyslipidemia is showing an increasing prevalence in T1DM, and the wealth of evidence gathered in recent years shows the significant contribution of serum lipid profiles to DPN. Although a variety of factors could be involved, the precise processes by which they may affect peripheral nerves in children with T1DM and their link remain unclear and contradictory [10–13].

According to reports, up to 50% of cases of DPN may not exhibit any symptoms [7]. Therefore, the presence of reliable and practical biomarkers that are related to the progression of DPN, together with clinical symptoms and neurological findings, may help in the early detection of the condition [14]. Neuron specific enolase (NSE) is well known as a tumor marker and is a measure of nerve damage. Studies have shown that disorders leading to comparatively rapid neuronal damage tend to have higher serum and cerebrospinal fluid levels of NSE. Additionally, its serum levels may be altered through degeneration and regeneration of peripheral nerves due to chronic hyperglycemia and associated oxidative stress [15]. Furthermore, various studies have investigated serum levels of a small protein called heat shock protein 27 (HSP 27), which is crucial for cells’ defense against stress in patients with diabetic neuropathy [16]. In experimental models of axon injury, HSP 27 plays a critical role in both axonal regeneration and neuron survival. Moreover, familial polyneuropathies can result from mutations in HSP 27 [17, 18]. Still, there is a dearth of information available regarding them in children with DPN.

Herein, guided by NCS, our goal was to assess the role of MNSI, lipid profile, serum HSP 27, and serum NSE in predicting asymptomatic DPN in children and adolescents with T1DM.

## Methods

A total of 63 children diagnosed with T1DM for at least 5 years were recruited from the Pediatric Endocrinology Clinic at Menoufia University Hospitals between July 2022 and June 2023. Patients previously diagnosed with PN related to diabetes or other reasons (e.g., vitamin B12 deficiency, other autoimmune diseases, infections, drugs, tumors, inherited disorders, or exposure to toxins) were excluded from the study. Thirteen children were also excluded as their caregivers rejected NCS, so 50 patients completed the study with 50 age- and sex-matched apparently healthy children as controls.

After obtaining an informed consent, all patients were subjected to a detailed clinical history emphasizing the age of onset and duration of T1DM, insulin regimen, adherence to therapy, and blood glucose monitoring methods. Anthropometric measurements such as height, weight, and body mass index (BMI) were recorded, and a complete physical and neurological examination was conducted. Assays for HbA1c, lipid profile, serum NSE, and HSP27 were measured for both patients and controls, and only patients were subjected to MNSI scoring and NCS.

MNSI includes both questionnaire and physical examination parts. A handful of the questions were translated to native language to make them more understandable for the study participants. Responses to the questionnaire by answering “yes” to questions 1–3, 5–6, 8–9, 11–12, 14–15 and “no” to questions 7 and 13 give one point for each question. Questions 10, “Do you feel weak all over most of the time?” and 4, “Do you get muscle cramps in your legs and/or feet?” were excluded from the published scoring methodology because they were deemed to represent measures of general asthenia and poor circulation, respectively [19, 20].

During the MNSI examination, each foot was inspected for deformities, dry skin, calluses, infections, and fissures, and given a score of 1 for any abnormality. The ankle reflex was also elicited and received a score of 0.5 if it tested positive with reinforcement and 1 if absent. Vibration sensation was tested by using a 128-Hz tuning fork over the great toe and received 0.5 if vibration was sensed for ≥ 10 s and 1 if absent. Using the 10-gm nylon monofilament placed gently on the child’s big toe while closing his eyes, light-touch perception was tested and scored 1 if failed to be detected at all and scored 0.5 if the child detected the filament in less than 8 out of 10 test repetitions [19, 20].

Patients in the current study underwent NCS on both sides for common peroneal, posterior tibial, and sural nerves. Peripheral neuropathy is considered when there are abnormalities in at least two of the electrophysiological parameters; action potential (AP) amplitude and conduction velocity falling below the third percentile value of the control, and distal latency being prolonged than the upper normal limit. These limits were 2 milivolt (mV), 44 m/second (m/sec) and 6.5 millisecond (ms) for the common peroneal nerve; 4 mV, 41 m/sec and 5.8 ms for the posterior tibial nerve; and 6 microvolt (µV), 40 m/sec and 4.4 ms for the sural nerve, respectively [21, 22].

In the laboratory evaluation, venous blood samples were withdrawn, and serum was separated under aseptic conditions. The AU 680 Beckmann autoanalyzer (Beckmann) was used for immediate assay of HbA1c, total cholesterol (TC), low-density lipoprotein cholesterol (LDL-C), high-density lipoprotein cholesterol (HDL-C), and triglycerides (TG). A serum aliquot was stored at − 20 °C until serum NSE and HSP27 were estimated by enzyme linked immunosorbent assay (ELISA) according to manufacturer’s protocol (SunRed, China).

### Statistical analysis

Normally and non-normally distributed quantitative data were presented as mean ± standard deviation and as median (interquartile range), respectively. When comparing means, the Mann-Whitney U test and t-test were used for non-normally and normally distributed data, respectively. Correlations were tested using Spearman and Pearson coefficients. Statistical significance was considered at a p-value ≤ 0.05. The receiver Operating Characteristic (ROC) curve was used to assess the discriminating performance of serum NSE, HSP27 and MNSI. Linear regression analysis was used to assess predictors of DPN in the studied patients. All analyses were performed using IBM SPSS Statistics for Windows, Version 20.0. (IBM Corp, Armonk, NY).

In accordance with a review of an earlier study [15], statistics and the Sample Size Pro tool version 6 determined the sample size to be at least 40 subjects, with 80% study power and 95% confidence level.

## Results

The mean age of studied patients with T1DM was comparable to that of the controls (13.70 ± 2.97 versus 13.68 ± 2.97 years). HbA1c, TC, TG, LDL-C, NSE and HSP27 showed a higher statistical significance in patients than in controls (Table 1). All diabetic children were on an intensive insulin regimen (basal and bolus doses through daily multiple injections), and none of them had continuous glucose monitoring through glucose sensors; only capillary blood glucose testing from a fingertip prick was used. Their mean HbA1c was 8.91 ± 1.17%.

**Table 1:** Comparison between clinical and laboratory data in patients and controls

Variables	Patients (*n* = 50)	Controls (*n* = 50)	*p*-value
**Sex: ** ***n*** **(%)**			
Male Female	19 (38.0%) 31 (62.0%)	20 (40.0%) 30 (60.0%)	0.838
**Age (years)**			
Mean ± SD	13.70 ± 2.97	13.68 ± 2.97	0.973
**Consanguinity n(%)**			
Present Absent	13 (26.0%) 37 (74.0%)	5 (10.0%) 45 (90.0%)	0.037*
**Weight z score**			
Mean ± SD	0.62 ± 0.53	0.56 ± 0.48	0.554
**Height z score**			
Mean ± SD	-0.13 ± 0.73	0.19 ± 0.74	0.032
**Body mass index z score**			
Mean ± SD	0.75 ± 0.58	0.59 ± 0.34	0.096
**HbA1c (%)**			
Mean ± SD	8.91 ± 1.17	4.57 ± 0.25	< 0.001**
**Total cholesterol (mg/dL)**			
Mean ± SD	155.74 ± 12.36	143.86 ± 11.23	< 0.001**
**Triglycerides (mg/dL)**			
Mean ± SD	96.96 ± 23.31	87.56 ± 9.92	0.011*
**LDL-C (mg/dL)**			
Mean ± SD	89.92 ± 13.97	78.68 ± 6.14	< 0.001**
**HDL-C (mg/dL)**			
Mean ± SD	57.14 ± 9.89	56.28 ± 9.34	0.656
**Serum neuron specific enolase (ng/ml)**			
Median (IQR)	36.45 (15.91–47.94)	11.98 (11.19–14.52)	< 0.001**
**Serum heat shock protein 27 (ng/ml)**			
Median (IQR)	59.43 (19.48–77.89)	18.69 (17.49–19.85)	< 0.001**

**HDL-C;** high density lipoprotein cholesterol, **IQR;** inter quartile range, **LDL-C;** low density lipoprotein cholesterol, **SD;** standard deviation, * Statistically significant at *p* ≤ 0.05, ** highly Statistically significant *p* < 0.001

**Fig. 1:**
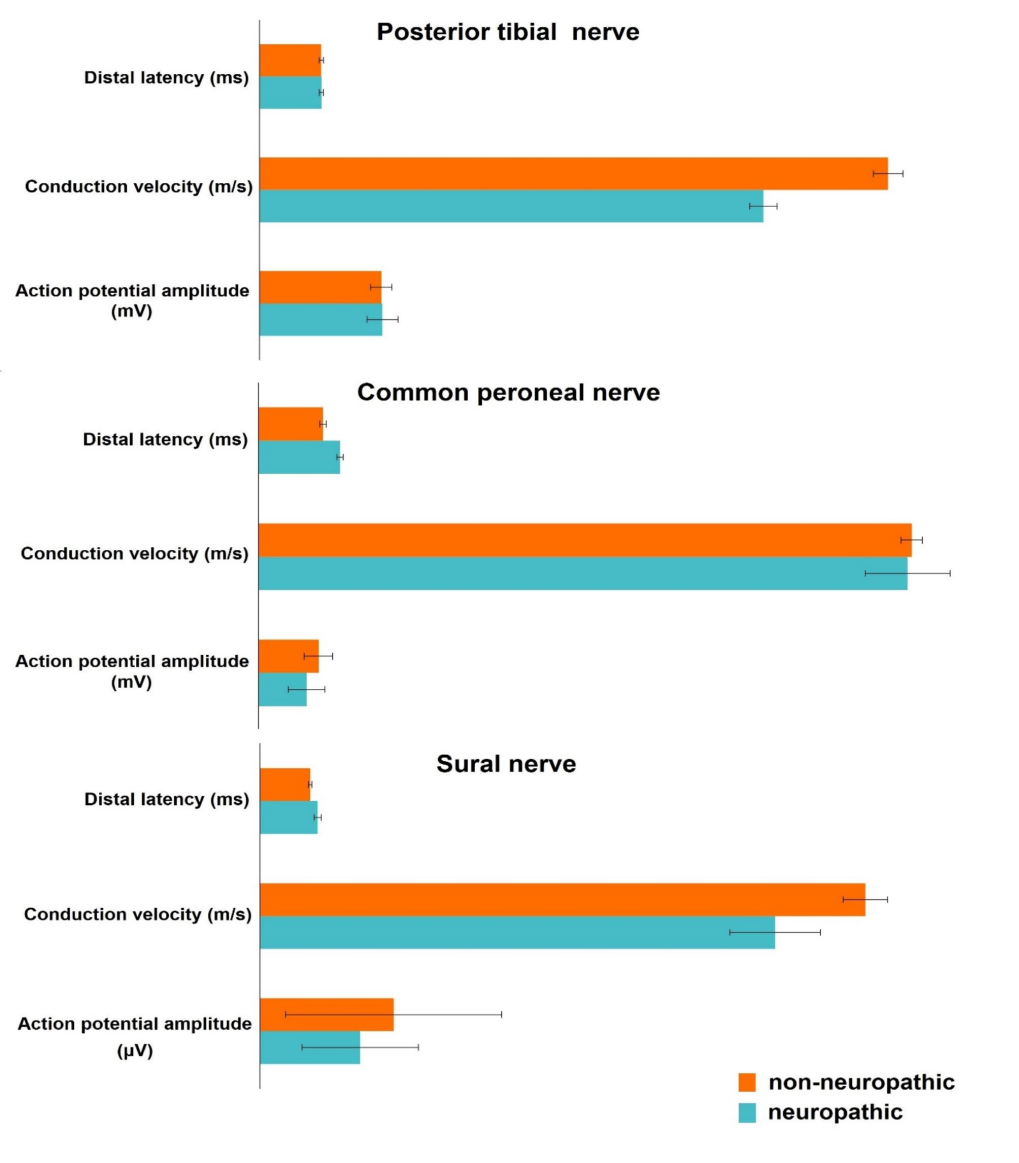
Electrophysiological changes in the studied peripheral nerves in neuropathic and non-neuropathic patients

The prevalence of DPN among the studied patients detected through NCS was 24% (12/50). Compared to non-neuropathic patients, neuropathic patients had statistically higher levels of HbA1c, TC, TG, LDL-C, NSE, and HSP27, as well as significantly older age, earlier age of diabetes onset, and longer disease duration. A higher BMI was also observed in neuropathic patients, despite being within normal ranges. Additionally, neuropathic patients had higher median MNSI questionnaire and examination score 5 and 2 compared to 4 and 0 in non-neuropathic patients, respectively. Furthermore, neuropathic patients had a marked reduction in sural AP amplitudes by NCS, with mild slowing of the conduction velocities of posterior tibial and sural nerves. There was also a statistically significant difference concerning the distal latencies of the common peroneal and sural nerves between neuropathic and non-neuropathic patients, but still within the normal range for age **(**Tables 2**and** Fig. 1**)**.

**Table 2:** Comparison between clinical data, laboratory data, Michigan neuropathy screening instrument and electrophysiological parameters in neuropathic and non-neuropathic patients

Variables	Neuropathic patients (*n* = 12, 24%)	Non-neuropathic patients (*n* = 38, 76%)	*p*-value
**Sex ** ***n*** **(%)**			
Male Female	5 (41.7%) 7 (58.3%)	14 (36.8%) 24 (63.2%)	1.000
**Age (years)** Mean ± SD	16.92 ± 0.90	12.68 ± 2.65	< 0.001**
**Age of onset of T1DM (years)** Median (IQR)	3.5 (3–7)	7 (4–10)	0.003*
**Disease duration (years)** Mean ± SD	9.17 ± 0.72	6.68 ± 1.58	< 0.001**
**Weight z score**	0.64 ± 0.55	0.62 ± 0.53	0.911
**Height z score**	-0.39 ± 0.62	-0.05 ± 0.74	0.157
**Body mass index z score**	0.96 ± 0.49	0.64 ± 0.43	0.035*
**HbA1c (%)** Mean ± SD	10.41 ± 0.49	8.43 ± 0.85	< 0.001**
**Total cholesterol (mg/dL)** Mean ± SD	166.75 ± 14.40	152.26 ± 9.43	0.006*
**Triglycerides (mg/dL)** Mean ± SD	109.83 ± 29.89	92.89 ± 19.58	0.027*
**LDL-C (mg/dL)** Mean ± SD	104.17 ± 17.49	85.42 ± 8.99	< 0.001**
**HDL-C (mg/dL)** Mean ± SD	61.42 ± 12.22	55.79 ± 8.79	0.160
**Serum neuron specific enolase (ng/ml)**			
Median (IQR)	51.05 (44.85–58.46)	33.35 (14.23–43.07)	< 0.001**
**Serum heat shock protein 27 (ng/ml)**			
Median (IQR)	82.70 (62.93–85.78)	49.94 (17.62–73.01)	< 0.001**
**MNSI questionnaire score** Median (IQR) **MNSI examination score** Median (IQR)	5 (4.25-6) 2 (1.13–2.5)	4 (4–5) 0 (0–0)	0.034* < 0.001**
**Sural nerve**			
Action potential amplitude (µV) Mean ± SD Conduction velocity (m/s) Mean ± SD Distal latency (ms) Mean ± SD	7.75 ± 4.51 39.83 ± 3.51 4.45 ± 0.28	10.34 ± 8.36 46.82 ± 1.71 3.88 ± 0.13	0.168 < 0.001** < 0.001**
**Posterior tibial nerve**			
Action potential amplitude (mV) Mean ± SD Conduction velocity (m/s) Mean ± SD Distal latency (ms) Mean ± SD	9.58 ± 1.21 39.25 ± 1.06 4.82 ± 0.15	9.49 ± 0.83 48.97 ± 1.15 4.81 ± 0.18	0.828 < 0.001** 0.946
**Common peroneal nerve**			
Action potential amplitude (mV) Mean ± SD Conduction velocity (m/s) Mean ± SD Distal latency (ms) Mean ± SD	3.69 ± 1.41 49.92 ± 3.26 6.26 ± 0.24	4.61 ± 1.11 50.24 ± 0.82 4.94 ± 0.24	0.060 0.576 < 0.001**

**HDL-C;** high density lipoprotein cholesterol, **IQR;** inter quartile range, **LDL-C;** low density lipoprotein cholesterol, **MNSI;** Michigan Neuropathy Screening Instrument, **mV;** millivolt, **µV;** microvolt, **m/s;** meter per second, **ms;** millisecond, **SD;** standard deviation, **T1DM;** type 1 diabetes mellitus, * Statistically significant at *p* ≤ 0.05, ** highly Statistically significant *p* < 0.001

Regarding correlations between the studied parameters, HbA1c exhibited a statistically significant positive correlation with TC, LDL-C, TG, HSP27, NSE, the MNSI questionnaire, the MNSI examination and sural nerve distal latency (*r* = 0.296; *p* = 0.037, *r* = 0.421; *p* = 0.002, *r* = 0.461; *p* = 0.001, *r* = 0.283; *p* = 0.046, *r* = 0.512; *p* < 0.001, *r* = 0.282; *p* = 0.047, *r* = 0.456; *p* = 0.001, *r* = 0.417; *p* = 0.003, *r* = 0.433; *p* = 0.002, respectively), and a statistically significant negative association with the conduction velocities of the sural, posterior tibial, and common peroneal nerves (*r*=-0.388; *p* = 0.005, *r*=-0.316; *p* = 0.025, *r*=-0.385; *p* = 0.006, respectively). Elevated TC, LDL-C, and TG levels were inversely correlated with sural conduction velocity (*r*=-0.316; *p* = 0.025, *r*=-0.560; *p* < 0.001, *r*=-0.281; *p* = 0.048, respectively).

**Fig. 2:**
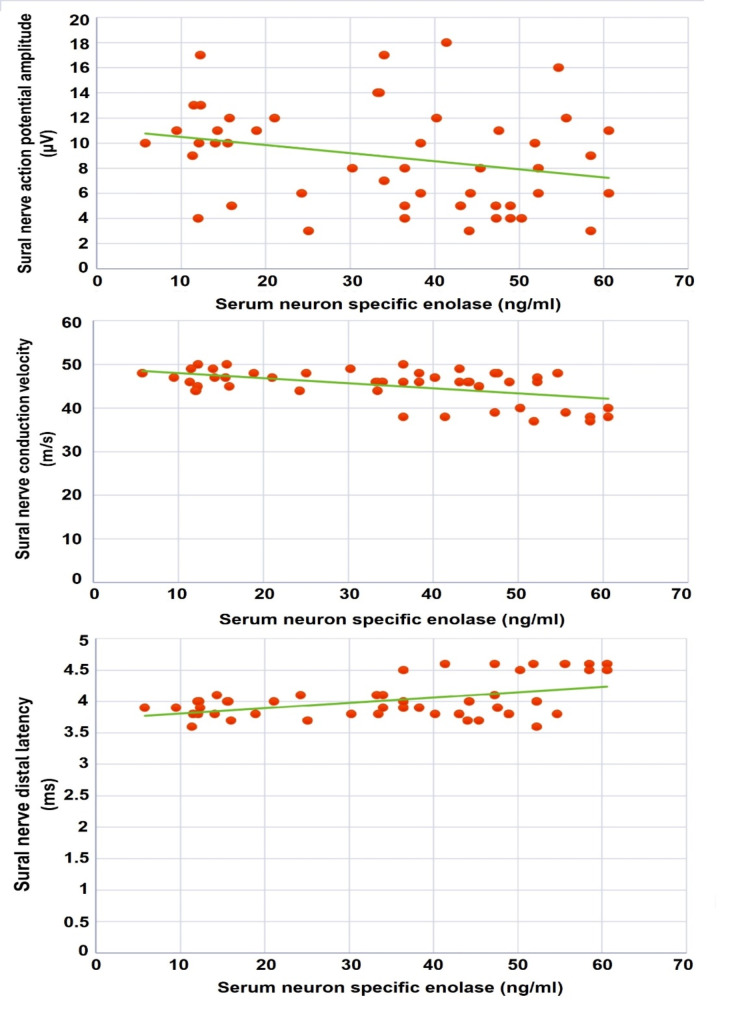
Correlations between serum neuron specific enolase levels and sural nerve electrophysiological parameters in the studied patients

NSE showed a statistically significant positive correlation with disease duration, HbA1c, HSP27, the MNSI questionnaire, the MNSI examination, and sural nerve distal latency (*r* = 0.387; *p* = 0.005, *r* = 0.512; *p* < 0.001, *r* = 0.542; *p* < 0.001, *r* = 0.342; *p* = 0.015, *r* = 0.496; *p* < 0.001, *r* = 0.311; *p* = 0.028, respectively), and a statistically significant negative correlation with sural AP amplitude and the conduction velocities of the sural, posterior tibial and common peroneal nerves (*r*=-0.285; *p* = 0.045; *r*=-0.404; *p* = 0.004, *r*=-0.331; *p* = 0.019, *r*=-0.280; *p* = 0.049, respectively) (Fig. 2). HSP27 had a statistically significant positive correlation with HbA1c, LDL-C, NSE, the MNSI questionnaire, and the MNSI examination (*r* = 0.283; *p* = 0.046, *r* = 0.311; *p* = 0.028, *r* = 0.542; *p* < 0.001, *r* = 0.337; *p* = 0.017, *r* = 0.474; *p* = 0.001, respectively), and a statistically significant negative correlation with sural AP amplitude (*r*=-0.333; *p* = 0.018).

**Fig. 3:**
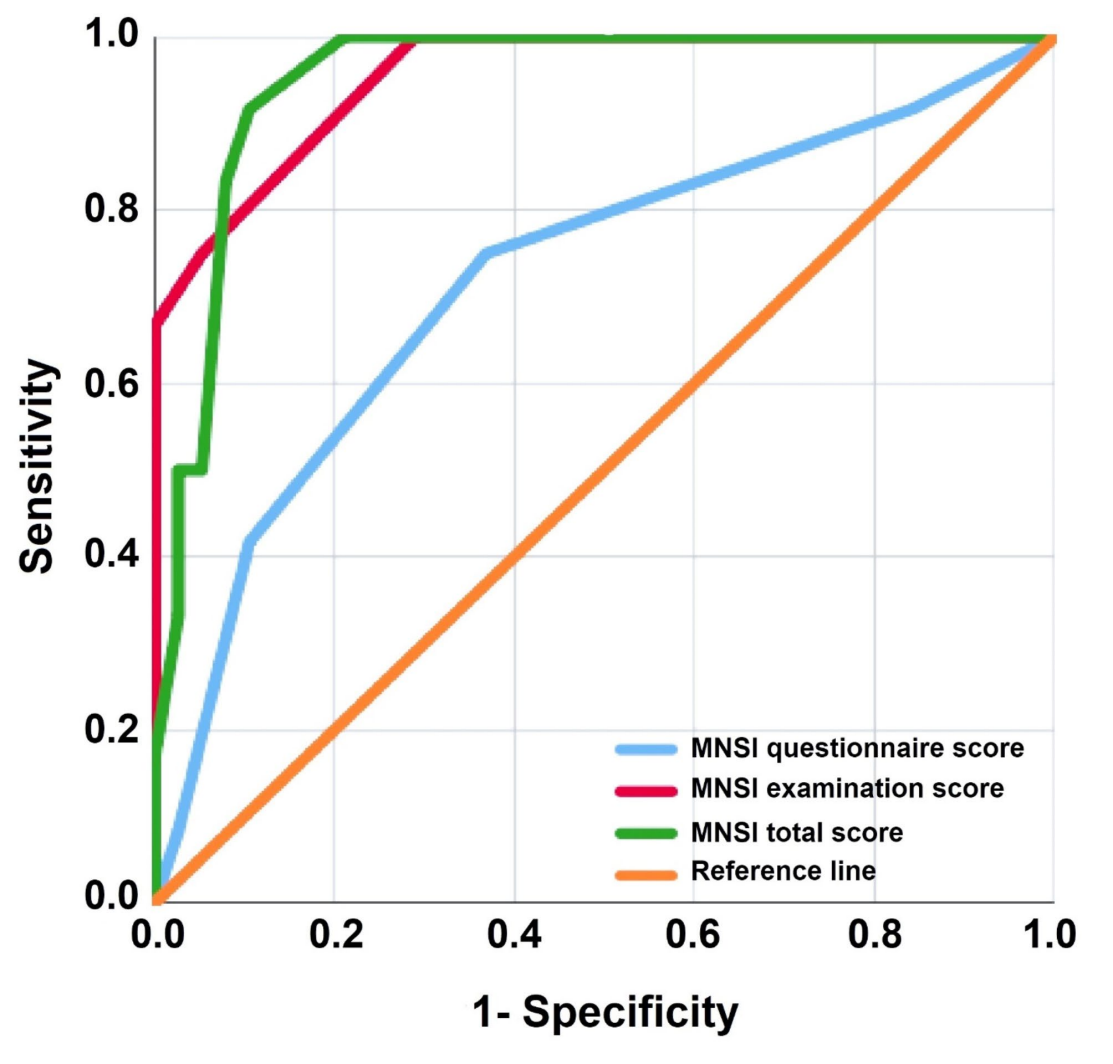
Receiver operating characteristic (ROC) curve for Michigan neuropathy screening instrument (MNSI) questionnaire, examination, and total scores to discriminate neuropathic patients from non-neuropathic ones

**Fig. 4:**
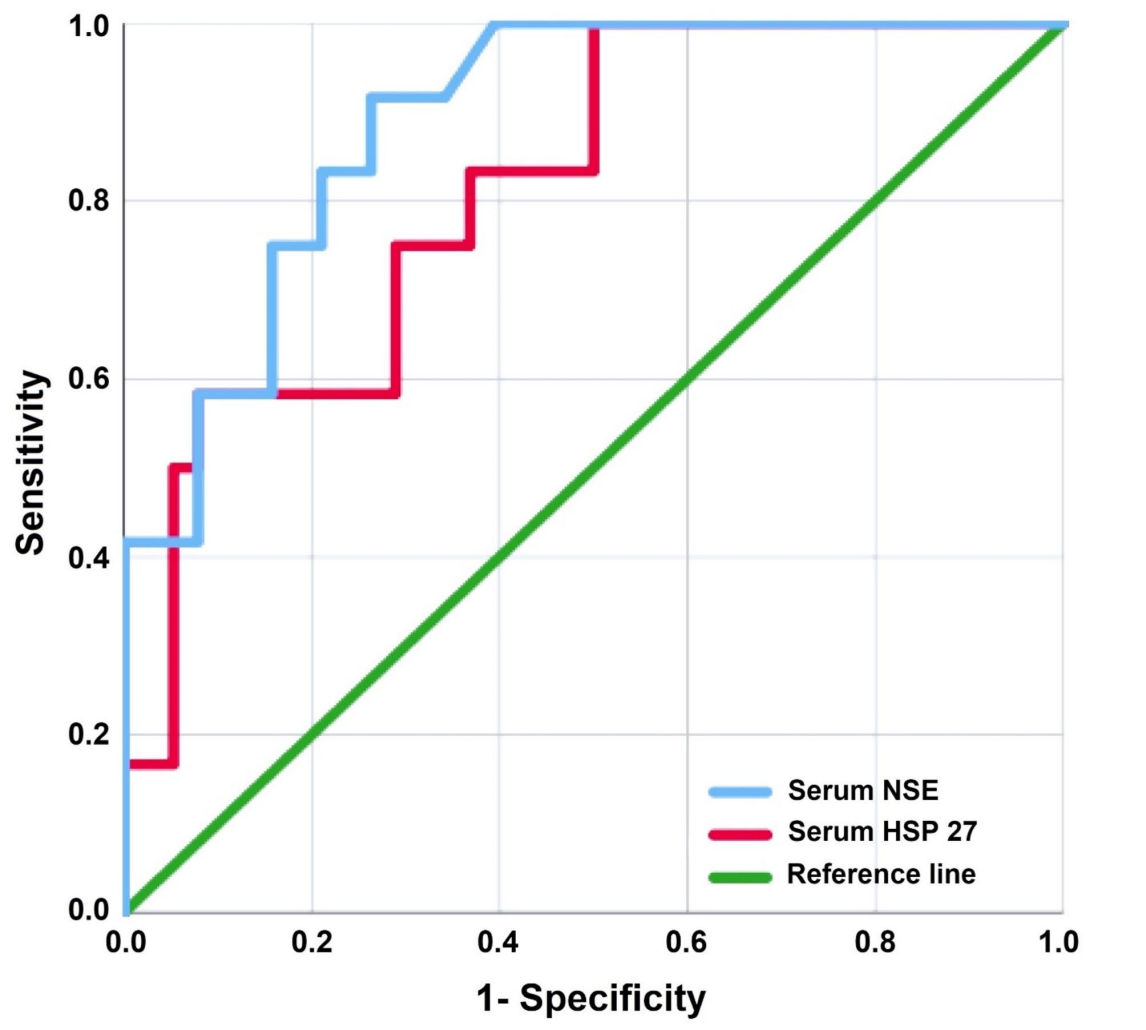
Receiver operating characteristic (ROC) curve for serum neuron specific enolase (NSE) and serum heat shock protein 27 (HSP 27) to discriminate neuropathic patients from non-neuropathic ones

With 75% sensitivity, 94.74% specificity, a 0.955 area under the curve (AUC), and *p* < 0.001, ROC analysis determined that a score of ≥ 1.5 for the MNSI examination was the best cutoff point for differentiating between neuropathic and non-neuropathic patients. For the MNSI questionnaire, the best cutoff point was ≥ 5, with 75% sensitivity, 63% specificity, a 0.720 AUC, and *p* = 0.002. Additionally, the entire MNSI score demonstrated 91.67% sensitivity, 89.47% specificity, a 0.951 AUC, and *p* < 0.001 at the optimal cutoff point of ≥ 6.5. Furthermore, compared to 66.92 ng/ml, a 0.814 AUC, 75% sensitivity, 71% specificity, and *p* < 0.001 for HSP27, NSE discriminated PN at a best cutoff point of 40.79 ng/ml with a 0.890 AUC, 92% sensitivity, 74% specificity, and *p* < 0.001 (Figs. 3 and 4).

Disease duration, HbA1c, TG, LDL-C, NSE, HSP27, and MNSI all showed a statistically significant association with DPN in univariate regression analysis. Nevertheless, in multivariate regression analysis, NSE and MNSI were the sole dependent predictors for DPN (Table 3).

**Table 3:** Univariate and multivariate regression analysis for detection of diabetic peripheral neuropathy predictors in the patients

Predictor	Univariable			Multivariable		
	cOR	95% CI	*p*-value	aOR	95% CI	*p*-value
**Disease duration (years)**	3.569	1.604–7.940	0.002*			
**MNSI questionnaire score**	2.259	1.120–4.555	0.023*			
**MNSI examination score**	54.0	7.818–372.984	< 0.001**	26.765	1.070–669.483	**0.045** ^*^
**MNSI total score**	5.001	1.925–12.997	0.001*	6.989	1.197–40.812	**0.031** ^*^
**HbA1c (%)**	26.517	2.669-263.462	0.005*			
**LDL-C (mg/dl)**	1.120	1.042–1.205	0.002*			
**Triglycerides (mg/dl)**	1.034	1.002–1.067	**0.034** ^*^			
**Serum heat shock protein 27 (ng/ml)**	1.051	1.015–1.089	0.005*			
**Serum neuron specific enolase (ng/ml)**	1.182	1.060–1.318	0.003*	1.200	1.002–1.438	**0.048** ^*^

**aOR;** adjusted odds ratio, **cOR;** crude odds ratio **CI;** Confidence interval, **LDL-C;** low density lipoprotein cholesterol, **MNSI;** Michigan Neuropathy Screening Instrument, * Statistically significant at *p* ≤ 0.05, ^**^ highly Statistically significant *p* < 0.001

## Discussion

Globally, the prevalence of both type 1 and type 2 diabetes is rising, and DPN is among the most distressing and costly chronic consequences of diabetes, leading to severe impairment and a poor quality of life, particularly in young individuals. DPN is frequently asymptomatic at early stages; however, once symptoms appear, there is no turning back. Thus, it is imperative to diagnose diabetic neuropathy early and take prompt action to stop its progression [23]. Our results reported DPN in 24% of the studied children evaluated by NCS, despite none of them having sought medical advice for DPN or having undergone a previous NCS.

NCS in T1DM exhibit diffuse alterations in a predictable manner that correlates with physical findings and long-term glycemic control, even in asymptomatic diabetic patients, in whom clinical examination may be less sensitive and specific [24]. However, it is laborious, costly, requires special expertise, and may possibly be declined by patients [8]. Moreover, the impairments in motor, sensory, and autonomic functions linked to DPN cannot be reliably assessed with a single clinical technique. Therefore, the ADA advised that DPN screening be carried out using a full clinical history, a comprehensive foot examination, a 10-g Semmes-Weinstein monofilament assessment, and at least one of vibration perception, pinprick, temperature perception, or ankle reflexes assessment. Thus, a systematic method to evaluate these deficits is medically necessary, especially in low-resource settings [25, 26].

Regarding this, many studies have highlighted the MNSI as a straightforward, non-invasive, and easy to use tool for assessing distal symmetrical PN in diabetic adults, with a sensitivity and specificity of up to 80% and 95%, respectively [2, 9, 20, 24, 27, 28]. Nevertheless, little research is available about the MNSI role as a screening tool for DPN in diabetic children. A systematic review by Franceschi et al. concluded that the MNSI is a useful practical tool with good sensitivity and specificity in detecting distal PN in children and adolescents, despite a lack of validation in these age categories [6]. Additionally, a recent study on diabetic adolescents highlighted that a positive MNSI questionnaire should raise concerns about possible early impairment to the sensory and motor nerve components [29].

Feldman et al. [19] determined a score ≥ 2.5 for the MNSI examination and a score ≥ 7 for the questionnaire to identify PN in diabetic adults. Later, Herman et al. [20] defined a new cutoff point ≥ 4 for the questionnaire, which improved the instrument efficiency. Concerning this, our findings highlighted that the MNSI examination score at ≥ 1.5 was a good specific test, with an AUC of 0.955, 94.74% specificity, and 75% sensitivity. On the other hand, the MNSI questionnaire showed the same sensitivity of 75% but lower specificity of 63%, with an AUC of 0.720 at the ≥ 5 cutoff point. This finding may be related to the questionnaire’s subjectivity and dependence on patients’ or their caregivers’ understanding of the required questions. Additionally, by combing both scores, overall sensitivity and specificity improved to 91.67% and 89.47%, respectively, with an AUC of 0.951. These results highlight the potential utility of MNSI in the screening of DPN in children and adolescents, but more research is required to validate and standardize its use in pediatrics.

Numerous studies have looked at the impact of hyperglycemia and dyslipidemia on DPN. The overall evidence continues to indicate that in T1DM, poor glycemic control and longer disease duration are associated with an increased risk of DPN. On the other hand, well-controlled hyperglycemia delays the development and progression of DPN, particularly when HbA1c values are below the 7.0% and 6.5% ranges, as advised by the ADA and the International Diabetes Federation, respectively [3, 30]. For dyslipidemia, oxidized LDL particles and free fatty acids may contribute to neuronal damage through generating reactive oxygen species, releasing pro-inflammatory substances, and adversely affecting healthy nerve myelination [31, 32]. Moreover, others have highlighted an upsurge association between poor glycemic control and increased levels of lipid peroxidation and oxidative stress in patients with T1DM, suggesting that poor glycemic control may be a potential modifiable risk factor for dyslipidemia [30, 33–35].

EURODIAB tracked adolescents and young adults with T1DM for eight years to identify incident DPN risk variables other than hyperglycemia. They found that increased levels of TC, LDL-C, TG, BMI, and hypertension were associated with DPN risk [36]. Additionally, a SEARCH study discovered that obesity, elevated TG, LDL-C, diastolic blood pressure, and decreased HDL-C were DPN risk factors [37]. In the same context, our results demonstrated a substantial relationship between elevated HbA1c levels and alterations in the lipid profile, with neuropathic patients showing significantly higher levels of TG, TC, and LDL-C, worse glycemic control, and a longer duration of disease than non-neuropathic patients. Furthermore, these variables showed significant correlations with the electrophysiological changes in the studied nerves, with some variations.

Nevertheless, others have reported that even with excellent metabolic control and a short disease duration, subclinical DPN has been detected in children with diabetes, indicating the possibility of genetic predisposition [3, 7, 38–40]. Additionally, wide variances in lipid profile across different studies have been reported, which could be attributed to variations in demographic profiles, including dietary and lifestyle habits, as well as influences of pubertal development [10, 11, 35]. As a result, their role in DPN remains contradictory and inapplicable to broader demographics. Even so, it is indispensable to keep children and adolescents with T1DM under adequate glycemic control and to monitor changes in their lipid profiles.

Biomarkers for diabetic neuropathy may arise from a variety of processes, such as degeneration of nerve fibers, damage to endothelial cells of neurotrophic blood vessels, and metabolic alterations that occur in peripheral nerve tissue under prolonged hyperglycemic conditions [41]. Serum NSE levels were found to be relatively elevated in diabetic adults and significantly elevated in those with diabetic neuropathy, and they may even be unaffected by age, diabetes type, disease duration, glycemic control, or the degree of neuropathy [15, 41–44]. Moreover, a recent study hypothesized that NSE might function as a predictive indicator for DPN therapeutic outcomes [45]. Few studies have reported elevated NSE levels in children with diabetic ketoacidosis [46, 47]; however, to the best of our knowledge, no other studies have examined the relationship between NSE and DPN in children with type 1 diabetes.

Heat shock proteins have been hypothesized to function as immunologic response modulators in type 1 diabetes, and their low expression may influence a number of diabetes-related pathological states and consequences [48]. Unfortunately, there is a dearth of contradictory data regarding HSP27 and DPN. Gruden et al. [16] reported a correlation between high serum HSP27 levels and DPN in type 1 diabetes; however, other studies found no such correlation [49, 50].

Several studies reported that the sural AP amplitude and conduction velocity, as well as peroneal conduction velocity threshold values, were the most effective indicators of diabetic neuropathy [51–53]. In this context, both NSE and HSP27 were significantly elevated in patients, and in relation to the studied electrophysiological changes, elevated NSE levels were linked to notable decreases in sural nerve AP amplitude and extensions in sural distal latency, along with decreased conduction velocities of the sural, common peroneal, and posterior tibial nerves, regardless of whether these electrophysiological changes remained within the normal range for age. On the other hand, elevated HSP27 levels were only correlated with the sural AP amplitude. Additionally, both levels distinguished DPN well in ROC analysis; however, serum NSE outperformed HSP27 in terms of sensitivity and specificity, suggesting that NSE is more relevant to DPN than HSP27.

From our findings, MNSI proved to be a sensitive and specific noninvasive clinical method for DPN screening. A longer disease duration, worse glycemic control, and significantly raised TC, TG, and LDL-C were all observed in neuropathic patients, with varying patterns of significance in relation to electrophysiological parameters; still, there are no clear statements regarding their relevance to DPN. Furthermore, serum NSE and HSP27 were significantly higher in patients than in controls, particularly in neuropathic patients. Despite poor data in pediatrics, NSE correlated better to electrophysiological changes than HSP27, showing superior sensitivity and specificity in DPN discrimination. In the same context, multivariate regression analysis revealed that MNSI and serum NSE were the only dependent predictors of DPN, surpassing disease duration, HbA1c, TG, LDL-C, and HSP27. This suggests that MNSI and serum NSE could be promising and valuable tools for evaluating DPN in children and adolescents with T1DM and may provide a trustworthy aid in clinical decision-making. Thus, we recommend carrying out further large-scale studies on them.

### Points of strength

As far as we know, our study is the first to assess DPN in children and adolescents with T1DM combining both MNSI, HbA1c, lipid profile, HSP27, and NSE, all under the guidance of the gold standard NCS. Additionally, none of the enrolled patients had ever sought medical advice for DPN or undergone a previous NCS.

### Limitations

The comparatively small sample size of this study was one of its primary limitations. Our staff also faced challenges when many parents refused to have NCS for their children, either out of fear of the test or because of societal beliefs about DM or a lack of medical knowledge. Additionally, the MNSI questionnaire was translated into our country’s native language, and scores were recorded based on the answers in the native language, ensuring that they conformed to the original questionnaire language; still, there is a risk of translation bias.

## Conclusion

In children with type 1 diabetes, DPN is a growing issue that requires attention and the development of practical screening tools to support subsequent interventions. MNSI is a feasible clinical tool that easy to use by medical personnel, especially in resource-limited settings. In addition, serum NSE levels correlate well with electrophysiological changes in DPN. Both tools show solid sensitivity and specificity in discriminating neuropathic patients according to our findings, underscoring their valuable role in this issue. Finally, even with the reported controversial data, it is crucial to follow up lipid profile in these children, especially with a longer duration of disease and poor glycemic control.

## Data Availability

The datasets used and/or analyzed during the current study are available from the corresponding author on reasonable request.

## Abbreviations

ADA: American Diabetes Association
AUC: Area under the curve
DM: Diabetes mellitus
DPN: Diabetic peripheral neuropathy
HDL-C: High-density lipoprotein cholesterol
HSP 27: Heat shock protein 27
LDL-C: Low density lipoprotein cholesterol
MNSI: Michigan neuropathy screening instrument
NSE: Neuron specific enolase
NCS: Nerve conduction study
PN: Peripheral neuropathy
ROC: Receiver Operating Characteristic
T1DM: Type 1 diabetes mellitus
T2DM: Type 2 diabetes mellitus
TC: Total cholesterol
TG: Triglycerides
